# Supplementary material for: Genetically engineered heat-resistant coral: An initial analysis of public opinion
Source: PLoS One. 2022 Jan 21;17(1):e0252739. doi: 10.1371/journal.pone.0252739 (PMC8782467; doi:10.1371/journal.pone.0252739)
Supplement: S1 File — (PDF) [file pone.0252739.s001.pdf]

SYNTHETIC BIOLOGY: Restoring the Great Barrier Reef

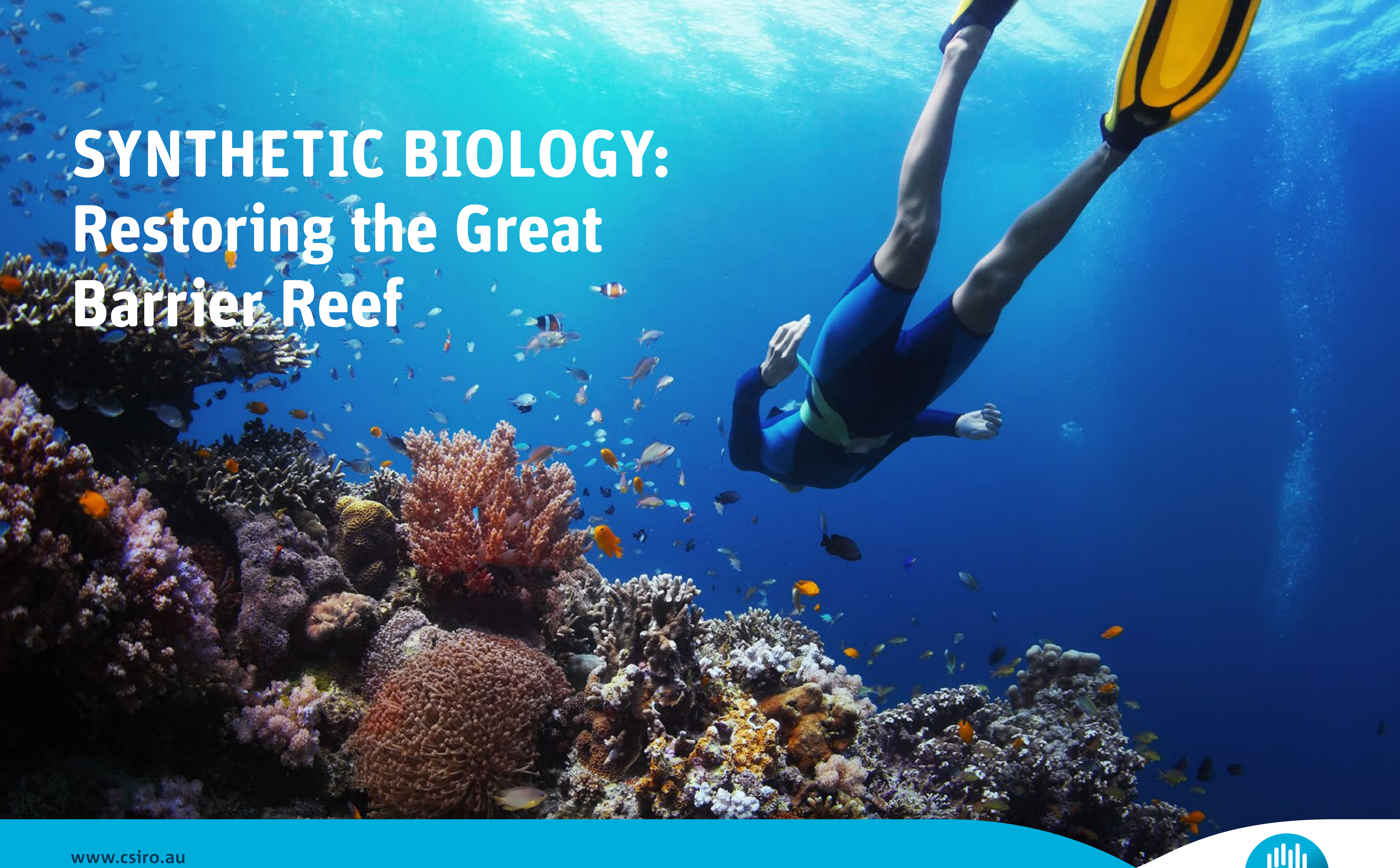

www.csiro.au

CSIRO

B&M | 18-00392

The Great Barrier Reef (GBR) in Australia is continuing to experience significant loss of coral, due to damage from:

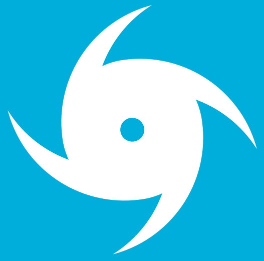

Tropical cyclones

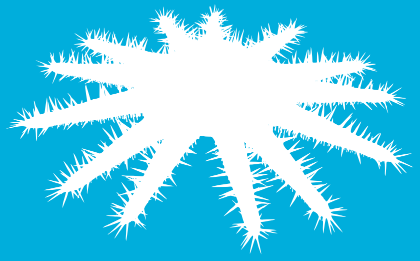

Crown-of-thorns starfish (which eat coral)

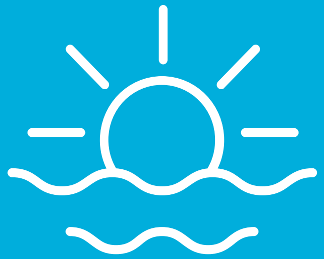

And high sea-surface temperatures (leading to recent mass coral bleaching events)

The loss of coral reef is a threat to fish and other marine animals that rely on coral for habitat and food.

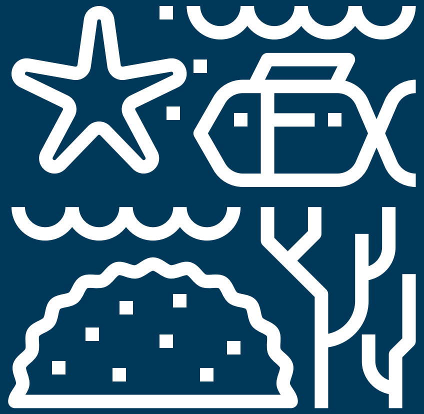

Damage to the Great Barrier Reef could also negatively impact fishers, tourist operators and others who depend on the reef for their livelihood.

Currently, manual methods are being used to protect the Reef from bleaching, such as:

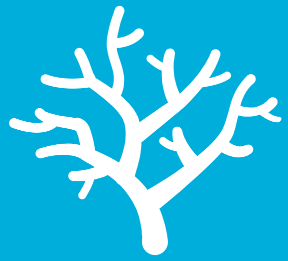

Manually restoring corals (farming)

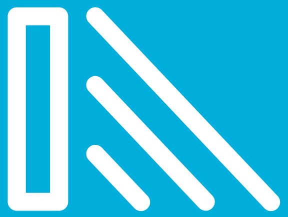

Shading and cooling techniques

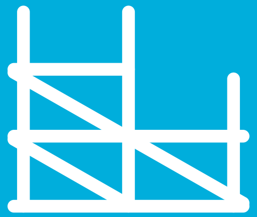

Reef structures and stabilisation

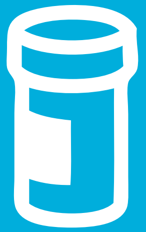

Probiotics to enhance survival during bleaching events

But these methods are not keeping up with the rate or scale of coral reef loss.

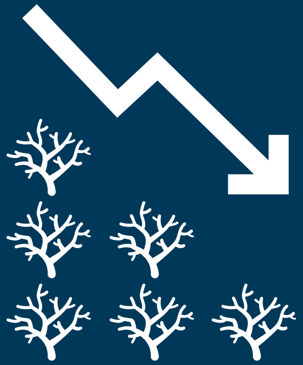

These methods are also labour-intensive, expensive and small-scale.

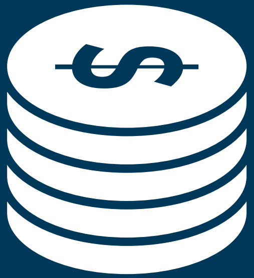

With new synthetic biology technology, it may be possible to genetically engineer existing coral species on the Great Barrier Reef to make them more tolerant to higher sea-surface temperatures.

This would help reduce the likelihood of coral bleaching.

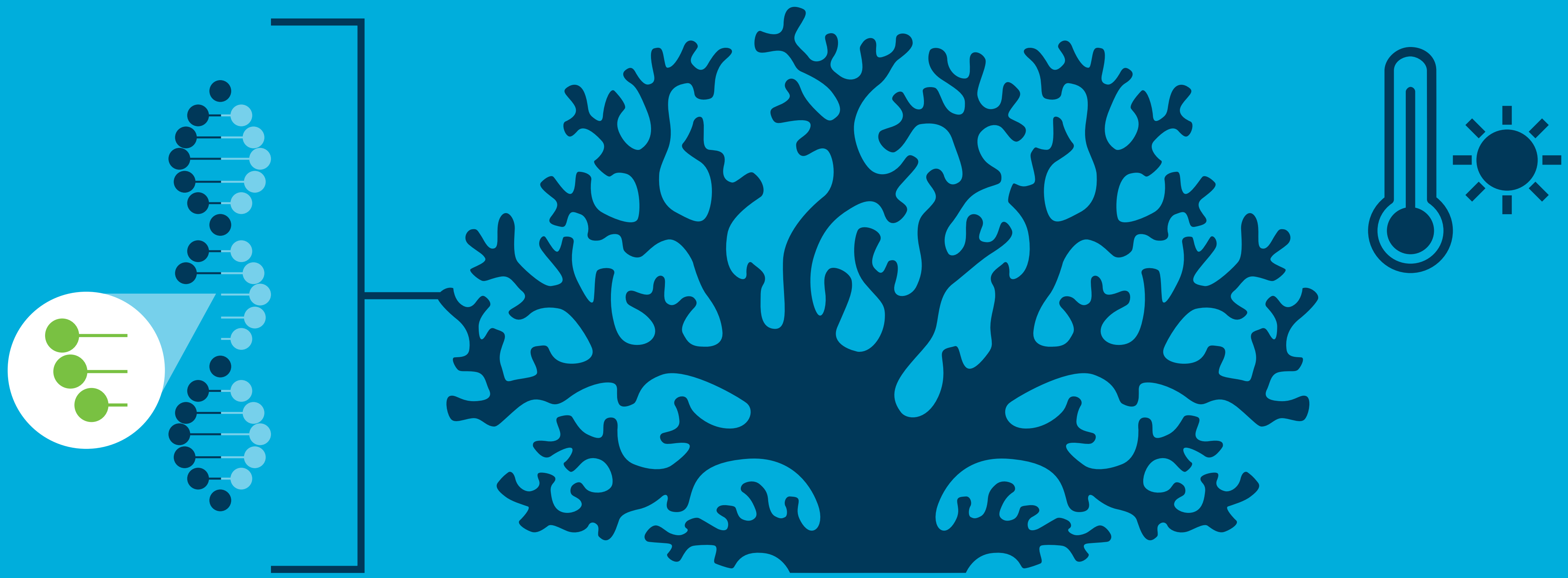

Lost or damaged coral in the Great Barrier Reef could be replaced with more resilient coral, potentially halting the rate of coral decline.

This could help to...

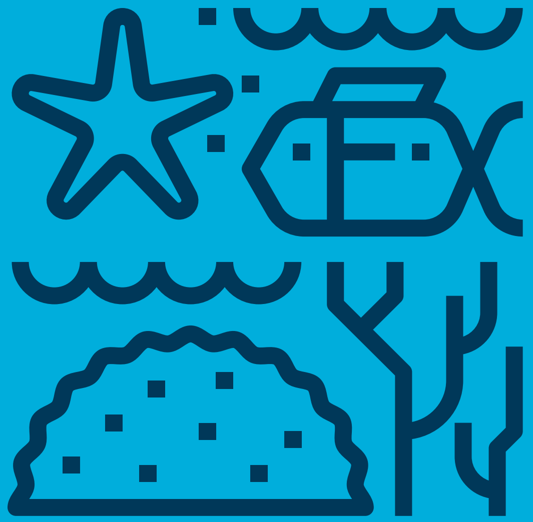

...restore coral habitat for fish...

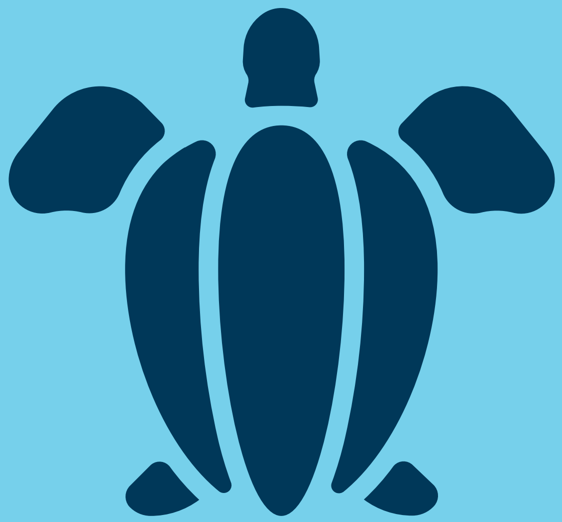

...and other marine animals...

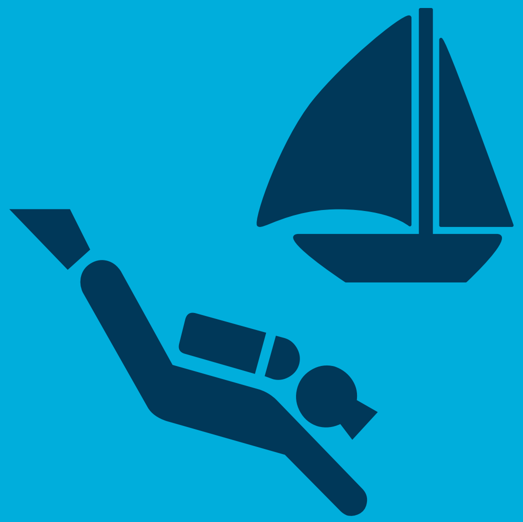

...and benefit local industries.

This technology would likely be approved and/or regulated by:

The **Office of the Gene Technology Regulator**

The **Environment Protection and Biodiversity Conservation Act**

The Great Barrier Reef Marine Park Authority

The State-based **Department of Fisheries**

Together these regulatory bodies and standards would ensure that:

- The research and development occurs under controlled laboratory conditions, and
- Any environmental, ecological and health risks or concerns are properly reviewed and addressed.

Australian residents like you may have the opportunity to ....

Take part in public events where scientists share their research on the technology

Participate in online or face-to-face discussions to ask questions and share your thoughts about the technology

Sign up to receive regular updates on the technology development

COPYRIGHT

© Commonwealth Scientific and Industrial Research Organisation 2018. To the extent permitted by law, all rights are reserved and no part of this publication covered by copyright may be reproduced or copied in any form or by any means except with the written permission of CSIRO.

IMPORTANT DISCLAIMER

CSIRO advises that the information contained in this publication comprises general statements based on scientific research. The reader is advised and needs to be aware that such information may be incomplete or unable to be used in any specific situation. No reliance or actions must therefore be made on that information without seeking prior expert professional, scientific and technical advice. To the extent permitted by law, CSIRO (including its employees and consultants) excludes all liability to any person for any consequences, including but not limited to all losses, damages, costs, expenses and any other compensation, arising directly or indirectly from using this publication (in part or in whole) and any information or material contained in it.

CSIRO is committed to providing web accessible content wherever possible. If you are having difficulties with accessing this document please contact us csiro.au/contact.
